# Differential participation in community cultural activities amongst those with poor mental health: Analyses of the UK *Taking Part* Survey

**DOI:** 10.1016/j.socscimed.2020.113221

**Published:** 2020-09

**Authors:** Daisy Fancourt, Louise Baxter

**Affiliations:** Department of Behavioural Science and Health, University College London, 1-19 Torrington Place, London, WC1E 7HB, UK

**Keywords:** Cultural engagement, Anxiety, Mental health, Community participation, Behaviour change theory

## Abstract

Rationale. There is a growing literature on the benefits of arts and cultural engagement for mental health. However, whether poor mental health is a barrier to engaging in cultural activities remains unclear. *Objective.* To identify whether there are differential participation rates in community cultural activities amongst those with differing levels of mental health (specifically, feelings of anxiety and happiness) and identify potential explanatory factors. *Method.* We analysed data from 7241 participants in the Taking Part survey; a random face-to-face household survey conducted in England (2016–2017). Cultural engagement was measured using a four-factor variable of cultural participation derived from assessing annual attendance at 21 receptive cultural activities. Mental health was measured using two of the Office for National Statistics measures of subjective wellbeing: happiness and anxious feelings. Analyses were adjusted for demographic, socio-economic, geographic and behavioural factors. *Results.* There was no difference in participation amongst individuals experiencing high levels of anxious feelings, but individuals experiencing low levels of happiness were less likely to engage in ‘popular’ cultural activities (e.g., live music events/cinema), ‘high art’ cultural activities (e.g., opera/ballet), and crafts and literary cultural events (e.g., exhibitions/book fairs). Education and socio-economic status largely explained differences, but for ‘high art’ and ‘popular’ activities, differences persisted independent of all explanatory factors tested. There was no difference in participation in global cultural activities (e.g., festivals). *Conclusions.* Using behaviour change theory, our findings suggest that lower levels of physical and social opportunity and psychological capability may reduce levels of cultural participation amongst individuals with low levels of happiness, but other physical and perceived barriers still remain to be explored.

## Highlights

• Low levels of happiness are associated with lower levels of cultural engagement.
• Education level and socio-economic position explain some of these differences.
• Mental health is not associated with participation in global cultural activities.
• High levels of anxious feelings are not associated with lower cultural engagement.

## 1 Introduction

There is growing research on the benefits of receptive cultural engagement for mental health (Fancourt and Finn, 2019). Attendance at cultural events, such as theatres, art galleries or museums, and cultural heritage sites has been found to (i) improve wellbeing and life satisfaction (Cuypers et al., 2012; Grossi et al., 2018; Meeks et al., 2017; Renton et al., 2012), (ii) reduce the risk of developing depression, and (iii) reduce anxiety and depression (Clements-Cortès, 2012; Fancourt and Tymoszuk, 2018). Still, not all people can access these cultural events, and, therefore, their potential benefits equally. There are substantially lower participation rates amongst individuals experiencing socio-demographic disadvantage (Arts Council England, 2013; Davies, 2015; Renton et al., 2012), individuals with low levels of educational qualifications (Arts Council England, 2013; Grisolía et al., 2010), older adults (Cuypers et al., 2012), people from minority ethnic groups, and those with a disability (Arts Council England, 2013; Potter, 2015).

Amongst those with poor mental health (e.g., low symptoms of hedonia and positive functioning) or with mental illness (e.g., symptoms relating to specific mental disorders), participation in activities such as exercise and socialising is frequently lower (Carpiniello et al., 2013; Glowacki et al., 2017). Additionally, some qualitative studies have reported perceived barriers to cultural engagement amongst individuals with mental illness, such as fear of being patronised (Jensen, 2018). But three clear questions remain: (i) whether levels of cultural engagement are significantly lower amongst those with poorer mental health, (ii) whether any potential differences are due to mental health itself or to those individuals being affected disproportionately by socio-economic barriers, and (iii) given research suggesting that socioeconomic factors differentially relate to different types of cultural engagement (Shariatinia, 2016), whether findings may differ depending on the type of cultural activity. These are important research questions given the reported benefits that cultural engagement for mental health and the clear strategic interest at organisational and policy levels in providing equality of access to cultural events. Therefore, this study explored differential participation in community cultural activities, focusing specifically on two aspects of mental health: feelings of anxiety and happiness.

## 2 Methods

### 2.1 Participants

Participants were drawn from the Taking Part Survey: a face-to-face household survey of a random representative sample of adults aged 16 or over living in England, commissioned by the Department for Culture, Media and Sport [dataset] (Department for Culture Media and Sport, 2016). We used data from Year 10 (2016–2017), which are the latest publicly available. Out of the 9352 adult participants surveyed, 2110 participants (22%) were missing data on covariates, so they were excluded from analyses. This left a total sample of 7241 participants for our main analyses.

### 2.2 Measures

Cultural engagement was measured using 21 variables measuring if participants had attended any of the following in the past 12 months in their own time: exhibition or collection of art, photography, or sculpture; craft exhibition; a video/electronic art event; books/writing event; street arts; public art display/installation; circus; carnival; culturally-specific festival; play or drama; pantomime; musical; opera/operetta; classical music performance; jazz performance; another live music event; ballet performance; contemporary dance performance; global dance performance; or another live dance event.

We focused on mental health, defined as a “syndrome of symptoms of hedonia and positive functioning, operationalised by measures of subjective well-being” (Keyes, 2005). Mental health is distinct from mental illness which refers to the presence of symptoms relating to specific mental disorders (Keyes, 2005). We measured two aspects of hedonic ‘experienced’ wellbeing: feelings of happiness and anxiety. We avoided focusing on aspects of evaluative or eudemonic wellbeing as this is already covered in broader literature (e.g., Steptoe and Fancourt, 2019). We used two of the Office for National Statistics (ONS) measures of personal well-being (Dolan et al., 2011): “overall, how happy did you feel yesterday” and “overall, how anxious did you feel yesterday”. Responses were from 0 (not at all) to 10 (completely). Given that there are no official cut-offs for these scales delineating good or poor mental health, and given both items show marked skew, we applied a range of thresholds in our analyses. As there are no official cut-offs for good or poor mental health using the ONS scales, for our main analyses, we defined poor mental health as being in approximately the worst 15% of responses for each measure, so we dichotomised the scales into ‘high anxious feelings’ (a score of ≥7; 14.3% of the sample) and ‘low happiness’ (a score of ≤5; 15.0% of the population). Sensitivity analyses tested different thresholds.

To identify whether any differences in participation were in fact due to some of the demographic factors identified as barriers in previous studies, we used measures of a range of demographic, socio-economic, geographic, and behavioural factors (see Supplementary Material).

### 2.3 Statistics

In order not to impose pre-conceived groupings on the different types of cultural engagement, we ran a preliminary factor analysis to identify latent groups using a matrix of tetrachoric correlations for all 21 binary items. The Kaiser-Meyer-Olkin measure of sampling adequacy was 0.91 (“marvellous”, indicating good suitability of data for factor analysis). Kaiser's criterion of eigenvalues >1 initially suggested a three-factor structure, but inspection of a scree plot proposed a four-factor structure and this was confirmed as a reasonable choice when considering the proportion of variance explained, for which four factors explained >5% (total cumulative proportion explained: 78%). Using both oblique and orthogonal rotations (excluding factors with a primary loading <.35) produced four identical factors. Factor 1 (“popular culture” included attending the cinema, circus, pantomime, musical, or live music event. Factor 2 (“high art culture”) included attending a play or drama, opera/operetta, classical music performance, or ballet performance. Factor 3 (“crafts and literary arts”) included attending a visual art exhibition, crafts exhibition, digital arts event, books/writing event, public art display/installation, or street arts. Factor 4 (“global arts”) included attending a carnival, culturally-specific festival, contemporary dance performance, global dance, or another live dance event.

We used χ^2^ tests to explore baseline demographic differences between groups and logistic regression analyses to explore differential rates of participation amongst those of high and low levels of anxious feelings and happiness. Baseline models controlled for age and sex. We explored which factors could explain differential participation rates by calculating the percentage of participatory difference (PPD) explained by the inclusion of potential explanatory variables (Lin et al., 1997), using the formula: PPD=(OR (E + C + X)–OR (E + C))/(1–OR (E + C))*100, where OR = odds ratio, E = exposure (arts engagement), C = baseline covariates (age and sex), and X = explanatory variables being tested. Analyses were weighted using random iterative weighting to match the sample to target population estimates for the UK.

Sensitivity analyses used multiple imputation by chained equations to increase sample size (logistic/multinomial/ordinal/linear regression according to variable type), generating 50 imputed datasets. The imputation model included all analysis variables including the exposure and outcome variables and increased the sample size to 9314. Further sensitivity analyses applied more lenient thresholds for poor mental health: the 20% most anxious feelings (≥6; 19.3% of respondents) or lowest happiness (≤6; 22.8% of respondents), and used the full linear scales. Finally, to disentangle mental health from physical health, we re-ran analyses excluding participants with poor self-rated health (*n* = 609). All models met regression assumptions and all analyses were conducted using Stata version 14.1 (Statacorp, Texas).

## 3 Results

### 3.1 Cultural engagement rates

All demographic statistic appear in Table 1. When adjusting just for age and sex, there was no difference in participation dependent on whether participants had anxious feelings ≥7 across any of the cultural activities (Table 2). However, those who showed low levels of happiness ≤5, were 46% less likely to engage in ‘popular’ cultural activities, 31% less likely to engage in ‘high art’ cultural activities, and 28% less likely to engage in crafts and literary cultural activities. For crafts and literary cultural activities, the largest explanatory factors were socio-economic status (39.3% explained), education (28.6%), and area deprivation (14.3%). In the fully-adjusted model, the relationship was attenuated. However, for both ‘popular’ and ‘high art’ cultural activities, although the factors explored did partially explain the relationship, the relationship was maintained in fully-adjusted models, with 36% of the association for ‘popular’ cultural activities and 19% of the association for ‘high art’ cultural activities unaccounted for by identified confounders. There was no difference in participation in global cultural activities.

**Table 1:** Demographic characteristics of the sample my self-reported anxious feelings and happiness.

Variable	Whole population (*n* = 7241)	High anxious feelings (anxious feelings≥7, *n* = 1033)	Low anxious feelings (anxious feelings<7, *n* = 6208)	Low happiness (happiness≤5, *n* = 1089)	High happiness (happiness>5, *n* = 6152)
Age					
16-24	4.3%	4.5%	4.2%	**4.22%**	**4.3%**
25-44	30.2%	28.0%	30.5%	**30.1%**	**30.2%**
45-64	35.6%	38.6%	35.1%	**38.3%**	**35.1%**
65-75	17.9%	16.0%	18.2%	**14.4%**	**18.5%**
75+	12.1%	12.9%	12.0%	**13.0%**	**12.0%**
Gender: female	53.2%	**57.5%**	**52.5%**	55.6%	52.8%
Ethnicity: white	91.9%	92.9%	91.8%	92.7%	91.8%
Nationality: British	92.9%	93.0%	92.9%	94.5%	92.7%
Educational attainment					
No qualifications	17.3%	**18.0%**	**17.2%**	**21.9%**	**16.5%**
GCSE/O levels	10.5%	**12.3%**	**10.2%**	**13.4%**	**10.0%**
A levels/trade apprenticeship	33.1%	**35.2%**	**32.7%**	**32.0%**	**33.3%**
Higher education/degree	39.2%	**34.5%**	**40.0%**	**32.8%**	**40.4%**
Socio-economic classification					
Higher managerial	40.0%	**35.4%**	**40.7%**	**32.6%**	**41.3%**
Intermediate	20.7%	**21.2%**	**20.6%**	**18.8%**	**21.1%**
Routine/manual	39.3%	**43.4%**	**38.7%**	**48.6%**	**37.7%**
Household income <£16,000	30.9%	**37.4%**	**29.8%**	**39.8%**	**29.3%**
Living in a couple	56.1%	53.8%	56.5%	**47.1%**	**57.7%**
Children in the household	27.1%	26.0%	27.3%	25.4%	27.4%
Working full-/part-time	55.0%	**48.9%**	**56.0%**	**49.5%**	**56.0%**
Self-rated health: bad/very bad	8.4%	**21.0%**	**6.3%**	**20.8%**	**6.2%**
Geographic location					
North	36.1%	**42.1%**	**35.1%**	**42.9%**	**34.9%**
Midlands	19.2%	**16.8%**	**19.6%**	**17.6%**	**19.4%**
East	10.8%	**9.7%**	**11.0%**	**9.6%**	**11.0%**
South	24.4%	**21.9%**	**24.8%**	**20.4%**	**25.1%**
London	9.5%	**9.5%**	**9.6%**	**9.6%**	**9.5%**
Urban dwelling	84.6%	85.1%	84.5%	86.5%	84.3%
High level of deprivation (IMD 1–3)	30.4%	**36.5%**	**29.4%**	**39.8%**	**28.8%**

Boldface: significant difference between groups (high vs low anxious feelings and low vs high happiness); p < .05.

**Table 2:** Logistic regression analyses of the associations between anxious feelings and low happiness and cultural engagement calculating the percentage of participatory difference explained by specific factors.

Explanatory Variable	Anxious feelings		Low happiness	
	Odds ratio (95% CI)	PPD^a^	Odds ratio (95% CI)	PPD
‘POPULAR’ CULTURAL ACTIVITIES				
Basic model (age, sex)	0.89 (0.74, 1.05)	**-**	**0.54 (0.45, 0.64)**	–
+ ethnicity and nationality	0.89 (0.74, 1.06)	0%	**0.53 (0.45, 0.64)**	−2.2%
+ education	0.90 (0.76, 1.08)	9.1%	**0.58 (0.49, 0.70)**	8.7%
+ socio-economic classification and income	0.97 (0.81, 1.16)	72.7%	**0.62 (0.52, 0.74)**	17.4%
+ marital status, children and employment status	0.94 (0.78, 1.12)	45.5%	**0.58 (0.48, 0.69)**	8.7%
+ region of residence, urban/rural dwelling	0.90 (0.76, 1.08)	9.1%	**0.55 (0.47, 0.66)**	2.2%
+ index of multiple deprivation	0.94 (0.79, 1.12)	45.5%	**0.58 (0.49, 0.69)**	8.7%
+ all	0.99 (0.83, 1.20)	90.9%	**0.64 (0.53, 0.77)**	21.7%
‘HIGH ART’ CULTURAL ACTIVITIES				
Basic model (age, sex)	0.87 (0.73, 1.03)	–	**0.69 (0.58, 0.83)**	–
+ ethnicity and nationality	0.87 (0.73, 1.03)	0%	**0.69 (0.58, 0.83)**	0%
+ education	0.90 (0.75, 1.08)	23.1%	**0.76 (0.63, 0.92)**	22.6%
+ socio-economic classification and income	0.89 (0.74, 1.06)	15.4%	**0.81 (0.67, 0.98)**	38.7%
+ marital status, children and employment status	0.93 (0.77, 1.13)	46.2%	**0.72 (0.60, 0.87)**	9.7%
+ region of residence, urban/rural dwelling	0.88 (0.74, 1.05)	7.7%	**0.71 (0.59, 0.85)**	6.5%
+ index of multiple deprivation	0.91 (0.76, 1.08)	30.8%	**0.74 (0.62, 0.89)**	16.1%
+ all	0.94 (0.78, 1.14)	53.8%	**0.81 (0.67, 0.99)**	38.7%
CRAFTS AND LITERARY CULTURAL ACTIVITIES				
Basic model (age, sex)	0.87 (0.73, 1.03)	–	**0.72 (0.61, 0.86)**	–
+ ethnicity and nationality	0.87 (0.73, 1.03)	0%	**0.72 (0.61, 0.86)**	0%
+ education	0.90 (0.75, 1.08)	23.1%	**0.80 (0.67, 0.97)**	28.6%
+ socio-economic classification and income	0.93 (0.78, 1.11)	46.2%	**0.83 (0.69, 0.99)**	39.3%
+ marital status, children and employment status	0.89 (0.75, 1.05)	15.4%	**0.74 (0.62, 0.89)**	7.1%
+ region of residence, urban/rural dwelling	0.88 (0.74, 1.04)	7.7%	**0.73 (0.61, 0.87)**	3.6%
+ index of multiple deprivation	0.90 (0.76, 1.06)	23.1%	**0.76 (0.64, 0.90)**	14.3%
+ all	0.92 (0.76, 1.11)	38.5%	0.84 (0.70, 1.01)	42.9%
GLOBAL CULTURAL ACTIVITIES				
Basic model (age, sex)	1.01 (0.82, 1.25)	–	0.85 (0.69, 1.05)	–
+ ethnicity and nationality	1.02 (0.83, 1.24)	1.0%	0.84 (0.68, 1.04)	−6.7%
+ education	1.03 (0.84, 1.26)	2.0%	0.88 (0.71, 1.09)	20%
+ socio-economic classification and income	1.03 (0.83, 1.26)	2.0%	0.87 (0.70, 1.08)	13.3%
+ marital status, children and employment status	1.03 (0.84, 1.27)	2.0%	0.87 (0.70, 1.08)	13.3%
+ region of residence, urban/rural dwelling	1.03 (0.84, 1.26)	2.0%	0.86 (0.70, 1.07)	6.7%
+ index of multiple deprivation	1.01 (0.82, 1.24)	0%	0.84 (0.68, 1.05)	−6.7%
+ all	1.05 (0.85, 1.28)	4.0%	0.89 (0.72, 1.10)	26.7%

a PPD = Percentage of participatory difference explained.

### 3.2 Supplementary analyses

Supplementary analyses found no material difference in results when using multiple imputation (Supplementary Table 1). When using a more lenient threshold, results were replicated, except that differences in participation in crafts and literary arts persisted independent of all identified confounders (Supplementary Table 2). When using linear mental health measures, results were the same for anxiety, and showed similar patterns for happiness, although due to the positive skew of the scale, these results should be interpreted with caution (Supplementary Table 3). When excluding those with poor self-rated health, results were materially unchanged (Supplementary Table 3).

## 4 Discussion

We found no differences in participation in community cultural activities amongst those with high levels of anxious feelings, but amongst those experiencing low levels of happiness, we found lower participation in ‘popular’ cultural activities (e.g., going to pantomimes, live music events, or the cinema), ‘high art’ cultural activities (e.g., going to the opera, classical concert, or ballet), and crafts and literary cultural events (e.g., exhibitions, art displays, and book fairs). While differences in education and socio-economic status explained the relationship for crafts and literary cultural activities, they acted only as a partial explanatory variable for ‘popular’ and ‘high art’ cultural activities, with differences persisting independent of further social and geographic factors.

Our findings can be understood through the lens of the COM-B behaviour change framework, which proposes that engagement in activities is influenced by our Capabilities, Opportunities and Motivations (COM-B) (Michie et al., 2011). Individual socioeconomic differences played an explanatory role in all three types of cultural activities for which there was a significant difference in participation, accounting for 17.4%–39.3% of the association, while area deprivation accounted for 8.7%–16.1% of the association. Socio-economic status and area deprivation can both affect *physical opportunity* to engage in activities (such as being unable to afford participation, lacking time to participate due to working/caring responsibilities, or not having activities available in the local area) (Michie et al., 2011). Previous studies have highlighted a gradient in cultural participation across levels of socioeconomic status (Cuypers et al., 2012; Grisolía et al., 2010; Renton et al., 2012); the same gradient found for those experiencing poor mental health (Allen et al., 2014). Thus, part of the participation differences could be due to adults experiencing low levels of happiness being disproportionately more likely to be of lower socioeconomic status and to live in more deprived areas. Similarly, education explained a large proportion of the difference in participation: 8.7% for ‘popular’, 22.6% for ‘high art’ and 28.6% for crafts and literary cultural activities. Education can affect *psychological capability* to engage (e.g., literacy skills and confidence) (Michie et al., 2011). This finding echoes other studies on education and cultural engagement (Davies et al., 2015; Grisolía et al., 2010; Renton et al., 2012), suggesting differences are partly explained by adults experiencing low levels of happiness being disproportionately more likely to have lower educational attainment.

Social factors played a minor explanatory role, with marital status, family composition and employment status accounting for 7.1%–9.7% of the association. Nevertheless, this suggests that differences in *social opportunity* may also offer some explanation for differential engagement levels. Research has shown that people with mental illness can experience feelings of exclusion (Dinos et al., 2004; Mann et al., 2017). Whilst our study focused on mental health rather than mental illness, it is possible that similar feelings (albeit perhaps operating at lower levels) were experienced by people with low levels of happiness and affected participation. Although arts activities can reduce these feelings (e.g., Gallant et al., 2019; Morse et al., 2015; Secker et al., 2011; Torrissen and Stickley, 2018), such feelings may act as an initial barrier to engagement. Individuals with low happiness may also have weaker social support networks around them and experience less of a sense of community connectedness or belonging (Terry et al., 2019). This sense of 'belonging' and 'community' has previously been shown to partially explain the relationship between community participation (which included cultural engagement) and psychological distress (Terry et al., 2019). Further, applying the concept of ‘sense of community’ may help elucidate the *lack* of difference in participation levels for ‘global cultural activities’. Many of these events, such as culturally-specific festivals, are embedded in membership of specific communities, engendering a greater feeling of social bonding, so even participants with low levels of happiness may still feel encouraged to have similar levels of participation to people with higher levels of happiness (Lee, 2013).

However, differences in ‘popular’ and ‘high art’ cultural activities amongst those with varying levels of happiness persisted independent of these socio-economic factors, suggesting that psychological capability or physical or social opportunity do not wholly explain findings. In considering why these differences persist, individuals with lower levels of happiness may have lower *reflective motivation* to engage, undertaking less explicit planning and setting fewer goals (Michie et al., 2011). The process of self-stigma (an internalising of cultural stereotypes) can also lead to ‘behavioural futility’ – the “why try” effect (Corrigan et al., 2016) – and thus, act as a barrier to help-seeking behaviours (Clement et al., 2015). Relatedly, low self-esteem and confidence amongst people with depression are widely reported (Sowislo and Orth, 2013), and this may extend to people with low levels of happiness. Feelings of futility and low self-esteem may mean that individuals feel unworthy of engaging or undertaking self-care. Indeed, qualitative work has reported feelings of being patronised amongst individuals with mental illness who engaged in museum activities (Jensen, 2018). These experiences could hinder explicit planning to participate.

People with low levels of happiness may also have blunted emotional responses to cultural activities and therefore gain less pleasure from engaging, affecting their *automatic motivation*. Indeed, individuals with mental illness have an impaired ability to regulate their emotions (Aldao et al., 2010; Compare et al., 2014; Joormann and Stanton, 2016), and can experience higher levels of anhedonia (Treadway and Zald, 2011). However, comparisons of emotional responses to arts engagement amongst those with and without depression have so far not shown marked differences (Fancourt and Ali, 2019). It also remains unclear in the literature whether differences in emotional respondes to cultural engagement could also affect motivations to engage in cultural activities.

Notably, we saw differences in participation amongst those with low levels of happiness but not high levels of anxious feelings. As one explanation, socioeconomic status may be less strongly linked with anxiety than it is with depression (Barbaglia et al., 2015; Mwinyi et al., 2017). As socio-economic status was the largest explanatory factor, it is possible that the social discrepancies underlying some of the findings relating to low happiness may be less pronounced amongst those experiencing anxious feelings. Indeed, the differences in demographic factors were smaller when comparing people with high and low anxious feelings than when comparing people with high and low levels of happiness. Alternatively, differences in the nature of anxious feelings and low happiness may account for the different results. The tripartite model of anxiety and depression proposes that both have shared components relating to non-specific symptoms of general distress, and specific components: physiologic arousal for anxiety and anhedonia for depression (Clark and Watson, 1991). Anhedonia has been discussed as a potential explanatory factor for the differences observed for people with low happiness but is less relevant to anxious feelings. Further, the physiological arousal component of anxiety may enhance emotional responses to cultural activities and actually support positive emotional feedback, supporting future attendance (Rickard, 2004). However, we specifically looked at a relatively general pool of participants: the 15% of the sample reporting highest levels of daily anxious feelings. It is likely that a more specific analysis of individuals with more anxiety-related mental *illness* (e.g., social anxiety disorder) may have different patterns of response and cultural engagement behaviours (Alden et al., 2008).

### 4.1 Strengths and limitations

Overall, this study has some significant strengths. It uses data collected from a nationally representative sample with a diverse list of cultural activities and rich data on covariates. However, this study focused specifically on self-reported feelings of happiness and anxiety rather than clinical measures of mental illness. Other research has shown a similar relationship between purpose and cultural engagement (Steptoe and Fancourt, 2019), but further research is needed on mental illness. In the absence of clear cut-offs on the ONS scales, we used 15th and 20th percentiles (finding consistent results across the two thresholds), but more research is needed looking at alternative ways of categorising mental health. We lacked a measure of physical health in our analyses, so it remains unclear whether comorbid physical health problems could have acted as physical barriers to participation. However, sensitivity analyses excluding those with ‘poor’ self-reported health confirmed our findings. Finally, due to limitations in the dataset we were only able to focus on potential explanatory variables relating to opportunities and capabilities. More research is needed to explore further barriers and enablers to participation, including those relating to motivations.

## 5 Conclusions

Overall, our analyses demonstrate a difference in participation in receptive cultural activities between people with low levels of happiness but not high levels of anxious feelings, suggesting that psychological factors relating to mental health could act as barriers to participatory behaviours. This difference is only partially explained by educational attainment, socio-demographic characteristics, and other social and demographic factors. Future studies should explore further the barriers to, and enablers of, cultural participation to support the development of interventions that could enable greater equality of participation, particularly amongst those who could benefit the most.

## Credit author statement

DF and LB designed the study. DF carried out the analyses. Both authors worked on the write up and critical appraisal of the manuscript and approved it for submission.
