# Supplementary material for: Differential participation in community cultural activities amongst those with poor mental health: Analyses of the UK Taking Part Survey
Source: Soc Sci Med. 2020 Sep;261:113221. doi: 10.1016/j.socscimed.2020.113221 (PMC7903156; doi:10.1016/j.socscimed.2020.113221)
Supplement: Multimedia component 1 [file mmc1.docx]

Supplementary Material

# Methods

To identify whether any differences in participation were in fact due to some of the demographic factors identified as barriers in previous studies, we used measures of a range of demographic, socio-economic, geographic and behavioural factors including gender, age (16-24, 25-44, 45-64, 65-74 and 75+), ethnicity (white vs other), nationality (British vs other), education (no qualifications, GCSE/O levels, A levels or trade apprenticeship, higher education/degree), socio-economic classification (based on NS-SEC SOC2010: higher managerial, intermediate or routine/manual), household income, marital status (living in a couple vs not living in a couple), whether there were any children living in the household, employment status (working full-/part-time vs not working), geographic location (government office region: North, Midlands, South, East or London), whether participants live in an urban or rural location, and level of deprivation where participants lived (using the Index of Multiple Deprivation). We also measured whether participants self-rated their general health as poor or very poor.

**Supplementary Table 1: Minimally and fully-adjusted logistic regression models showing associations between anxious feelings and low happiness and cultural engagement using multiple imputation**

| *N*=9,352 | Anxious feelings | Low happiness |
| --- | --- | --- |
|  | OR (95% CI) | OR (95% CI) |
| ‘Popular’ culture  Minimally adjusted  Fully adjusted | **0.83 (0.71, 0.97)**  0.92 (0.78, 1.09) | **0.55 (0.48, 0.64)**  **0.64 (0.54, 0.76)** |
| ‘High art’ culture  Minimally adjusted  Fully adjusted | 0.91 (0.78, 1.07)  1.00 (0.84, 1.19) | **0.74 (0.63, 0.88)**  0.88 (0.73, 1.04) |
| Crafts and literary arts  Minimally adjusted  Fully adjusted | 0.88 (0.75, 1.03)  0.94 (0.79, 1.11) | **0.74 (0.63, 0.86)**  0.85 (0.72, 1.00) |
| Global arts  Minimally adjusted  Fully adjusted | 0.99 (0.82, 1.20)  1.02 (0.85, 1.25) | 0.91 (0.75, 1.11)  0.97 (0.80, 1.18) |

**Supplementary Table 2: Minimally and fully-adjusted logistic regression models showing associations between anxious feelings and unhappiness and cultural engagement applying more lenient thresholds for anxious feelings and unhappiness**

| *N* = X,XXX | Anxious feelings≥6 | Happiness≤6 |
| --- | --- | --- |
|  | OR (95% CI) | OR (95% CI) |
| ‘Popular’ culture  Minimally adjusted  Fully adjusted | **0.83 (0.71, 0.97)**  0.92 (0.78, 1.09) | **0.66 (0.57, 0.76)**  **0.74 (0.63, 0.86)** |
| ‘High art’ culture  Minimally adjusted  Fully adjusted | 0.87 (0.73, 1.03)  0.94 (0.78, 1.14) | **0.77 (0.66, 0.90)**  **0.84 (0.71, 0.98)** |
| Crafts and literary arts  Minimally adjusted  Fully adjusted | 0.87 (0.73, 1.03)  0.92 (0.76, 1.11) | **0.79 (0.68, 0.91)**  **0.85 (0.73, 0.99)** |
| Global arts  Minimally adjusted  Fully adjusted | - 1. (0.82, 1.25)   1.05 (0.85, 1.28) | 0.89 (0.75, 1.05)  0.90 (0.76, 1.07) |

**Supplementary Table 3: Minimally and fully-adjusted logistic regression models showing associations between anxious feelings and low happiness and cultural engagement using linear mental health variables**

| *N* = X,XXX | Anxious feelings | Unhappiness |
| --- | --- | --- |
|  | OR (95% CI) | OR (95% CI) |
| ‘Popular’ culture  Minimally adjusted  Fully adjusted | 1.00 (0.98, 1.02)  1.01 (0.99, 1.04) | **0.93 (0.90, 0.96)**  **0.95 (0.92, 0.98)** |
| ‘High art’ culture  Minimally adjusted  Fully adjusted | 0.99 (0.97, 1.01)  0.99 (0.97, 1.02) | **0.96 (0.93, 0.99)**  0.97 (0.94, 1.00) |
| Crafts and literary arts  Minimally adjusted  Fully adjusted | 1.00 (0.98, 1.02)  1.00 (0.98, 1.02) | **0.97 (0.94, 1.00)**  0.98 (0.95, 1.01) |
| Global arts  Minimally adjusted  Fully adjusted | 1.00 (0.97, 1.02)  1.00 (0.97, 1.02) | **0.96 (0.92, 0.99)**  **0.96 (0.92, 1.00)** |

**Supplementary Table 4: Minimally and fully-adjusted logistic regression models showing associations between anxious feelings and unhappiness and cultural engagement excluding those with poor self-rated health**

| *N*=6,632 | Anxious feelings | Low happiness |
| --- | --- | --- |
|  | OR (95% CI) | OR (95% CI) |
| ‘Popular’ culture  Minimally adjusted  Fully adjusted | 1.00 (0.82, 1.22)  1.07 (0.87, 1.32) | **0.61 (0.50, 0.74)**  **0.68 (0.55, 0.84)** |
| ‘High art’ culture  Minimally adjusted  Fully adjusted | 0.89 (0.74, 1.08)  0.93 (0.76, 1.14) | **0.73 (0.60, 0.89)**  0.82 (0.77, 1.01) |
| Crafts and literary arts  Minimally adjusted  Fully adjusted | 0.90 (0.75, 1.08)  0.92 (0.75, 1.12) | **0.77 (0.64, 0.94)**  **0.87 (0.71, 1.06)** |
| Global arts  Minimally adjusted  Fully adjusted | - 1. (0.80, 1.26)   1.02 (0.82, 1.27) | 0.90 (0.71, 1.13)  0.92 (0.73, 1.15) |
